# An Assessment of Open Fracture Management in Hospitals in Malawi Before and Immediately After Implementing Open Fracture Guidelines

**DOI:** 10.2106/JBJS.OA.23.00078

**Published:** 2024-04-03

**Authors:** Maureen Sabawo, Zahra Jaffry, Linda Chokotho, Alexander Thomas Schade

**Affiliations:** 1 Kamuzu University of Health Sciences, Blantyre, Malawi; 2 Barts Health NHS Trust, The Royal London Hospital, London, England; 3 Malawi University of Science and Technology, Mikolongwe, Malawi; 4 Malawi-Liverpool-Wellcome Trust Clinical Research Programme, Blantyre, Malawi; 5 Liverpool School of Tropical Medicine, Liverpool, England

## Abstract

**Background:**

Open fractures, a common consequence of road traffic collisions, are associated with a high risk of complications. The introduction of standard guidelines has been shown to improve patient care and reduce the risk of complications in several countries. In September 2021, the Malawi Orthopaedic Association/Arbeitsgemeinschaft für Osteosynthesefragen Alliance (MOA/AOA) guidelines and standards for open fracture management were introduced in Malawi. This study aimed to assess the management of open fractures in hospitals in Malawi, before and after implementing a training course on the MOA/AOA open fracture guidelines.

**Methods:**

This was a descriptive and quantitative, before-and-after study that reviewed the medical files of patients with open fractures at Zomba Central Hospital and Mulanje, Salima, and Mangochi district hospitals over two 3-month periods. Variables included initial assessment; antibiotic prophylaxis; place of debridement; type of anesthesia; treatment of the open fracture in the emergency department, operating room, and wards; and short-term complications requiring hospital treatment.

**Results:**

A total of 88 open-fracture case files were reviewed; 43 were prior and 45 were subsequent to the implementation of the open fracture guidelines. The overall median patient age was 36 years (interquartile range, 27 to 45 years), and 91% (80) were male. Limb neurovascular status assessment and documentation improved from 26% (11) of the patients before the guidelines to 62% (28) afterward (p = 0.0002). The percentage who underwent debridement in the operating room significantly increased from 19% (8) to 69% (31) (p = 0.01). The percentage who underwent debridement under general or spinal anesthesia significantly increased from 5% (2) to 38% (17) and from 12% (5) to 29% (13), respectively (p= 0.001). The wound infection rate decreased from 21% to 11%, but this was not significant, and there was no change in the overall complication rate (p = 0.152).

**Conclusions:**

This study suggests that training on the MOA/AOA open fracture management guidelines followed by their implementation can lead to at least temporary improvement in the management of open fractures. Nevertheless, additional studies need to be performed to understand the effect on long-term patient outcomes.

**Levels of Evidence:**

Therapeutic Level III. See Instructions for Authors for a complete description of levels of evidence.

Injuries play a substantial role in the global disease burden, accounting for approximately 19.8% of disability-adjusted life years (DALYs) lost and causing 4.8 million deaths annually^1^. Notably, these deaths surpass the combined mortality figures for HIV/AIDS (human immunodeficiency virus/acquired immunodeficiency syndrome), tuberculosis, and malaria, and injuries are the leading cause of death among males aged 15 to 29 years^2-5^. Low and middle-income countries (LMICs) bear the brunt of this burden, having 90% of all deaths due to road traffic collisions (RTCs)^3^.

In Malawi, the average DALY loss due to RTCs was 180,000 per year between 2010 and 2020^6,7^. A substantial portion of fractures in Malawi, estimated at 66.5%, result from RTCs, while falls account for 16%^6^. The injured parties in RTCs are mainly pedestrians (32%), cyclists (28%), and motorcyclists (17.8%)^6,8^.

Open fractures, which are a common consequence of RTCs and falls, have an incidence of 30 per 100,000 person-years in high-income countries^3,4^. These fractures are orthopaedic emergencies that need to be prioritized because they are associated with an 18% risk of infection and 15% risk of amputation in LMICs^9^. In most LMICs, only 20% of patients with open fractures return to work within 1 year after the injury, resulting in far-reaching consequences for national economies, costing LMICs as much as 3% of their annual gross domestic product^3,9^. However, evidence suggests that high-quality open-fracture management is associated with improved outcomes and quality of life^10-13^. Therefore, many evidence-based guidelines have been formulated to improve the complex management of open fractures^11,14-17^.

Malawi is geographically divided into 4 regions: southern, northern, central, and eastern. The treatment of fractures in Malawi is provided through a 2-tiered health-care system. The secondary level of care is delivered by 117 orthopaedic clinical officers (OCOs) at 28 district hospitals distributed across the country. OCOs are non-physicians with a diploma in clinical orthopaedics or a bachelor’s degree in trauma and orthopaedics who provide nonoperative care for orthopaedic conditions as well as emergency orthopaedic surgery for select cases such as open fractures, dislocations, and acute infections^18^. Subsequently, more specialized care is offered at the country’s 4 tertiary hospitals by 9 specialized trauma and orthopaedic surgeons. Each tertiary hospital is situated in 1 of the 4 major cities within the respective regions mentioned earlier^18-20^.

Orthopaedic surgeons are specialist doctors with 4 years of postgraduate training, and they provide operative care for all orthopaedic and trauma conditions^18^. Plastic surgeons play a crucial role in providing reconstructive surgery to patients with traumatic injuries^18,20^. However, staffing of plastic surgeons at the trauma units at both levels remains minimal^19^. Only 2 plastic surgeons are currently available; both are situated in burn units within tertiary hospitals, and neither is situated at the recruitment hospitals of this study^21^.

As in many LMICs, the burden of injuries in Malawi is rising at a time when its health-care system has inadequate infrastructure and equipment and a shortage of personnel to treat injuries, especially open fractures, in both pre-hospital and in-hospital settings^19,22-24^.

In September 2021, locally adapted Malawi Orthopaedic Association/Arbeitsgemeinschaft für Osteosynthesefragen Alliance (MOA/AOA) guidelines were implemented through a training course. These guidelines recommend a primary Advanced Trauma Life Support (ATLS) survey, assessment and documentation of neurovascular status, early administration of antibiotic prophylaxis (within 1 hour after hospital presentation), thorough surgical wound debridement in the operating room under general or spinal anesthesia, proper bone stabilization, and early soft-tissue wound coverage to optimize outcomes^25^.

However, there is insufficient evidence from hospitals in Malawi to evaluate the management of open fractures and assess whether it is in line with these newly developed MOA/AOA guidelines. Thus, the aim of the present study was to assess the management of open fractures in hospitals in Malawi before the training course on the MOA/AOA guidelines compared with immediately afterward.

## Materials and Methods

### Study Design and Participants

This was a descriptive and quantitative, before-and-after study reviewed the medical files of patients with long-bone, hindfoot, and midfoot open fractures (involving the humerus, radius, ulna, femur, tibia, fibula, calcaneus, talus, tarsals, and metatarsals) treated from July 2021 to mid-February 2022. All patients who presented or were admitted to the 4 participating hospitals from July 2021 to mid-February 2022 and had radiographic confirmation of an open fracture of a long bone or of the hindfoot or midfoot were eligible for inclusion in the study. Patients with an open fracture of the hand or forefoot were excluded from the study^25^. The participating hospitals were Zomba Central Hospital and Salima, Mangochi, and Mulanje district hospitals.

The study protocol was approved by the College of Medicine Research Ethics Committee (P.08/21/3369) and by each participating hospital’s research management committee. Because we collected anonymous secondary data, individual consent was not required.

All data collection commenced at the same time, on November 15, 2021. Case files from July to September 2021 were reviewed retrospectively, and those from November 15, 2021, to February 15, 2022, after the training course that was held in September 2021, were reviewed prospectively.

### Data Collection

A data capture form was used to gather information from patient medical files and register books (see Appendix 1). This information included demographics as well as details regarding the initial assessment (including a primary survey and neurovascular status); antibiotic prophylaxis; temporary fracture immobilization; where the debridement was performed; type of anesthesia; soft-tissue wound closure and definitive treatment of the fracture in the emergency department, operating room, and wards; short-term complications requiring hospital treatment; and date and time of referral, treatment before referral, and reason for referral.

Data clerks recorded the demographics, disposition, and discharge and/or referral details, and OCOs recorded the clinical information in the initial management and definitive management sections. The principal investigator (PI) completed the complication section and verified each item of information captured by the data clerks and OCOs against the radiographs and wound photographs, to determine whether the patient treatment was consistence or inconsistent with the guidelines^26^. The PI also assessed wound infection using the ASEPSIS scoring system^25,27^. At the end of each month, the PI entered the completed and verified data into an electronic database.

### Definitions and Outcome Ascertainment

The collected data consisted of the mechanism of injury (RTC, fall from a standing height or above, sports, other), initial assessment and treatment in the emergency department (primary survey using ATLS protocol, neurovascular assessment, antibiotics, temporary immobilization), management in the operating room (place of debridement, type of anesthesia), definitive treatment (plaster of Paris [POP], external fixation, internal fixation, straight arm traction, skeletal traction, and amputation), management in the ward (timing of antibiotic administration, duration of antibiotics, tetanus toxoid vaccination status, wound care), and outcomes (including short-term complications requiring hospital treatment, such as wound infection, compartment syndrome, and amputation)^27^ (see Appendix 1).

### Statistical Analysis

Data were analyzed quantitatively using RStudio (R Foundation for Statistical Computing)^28^. Categorical demographic variables are presented as percentages and frequencies. Continuous variables are presented as the mean and standard deviation (for parametric data) or median and interquartile range (IQR) (for nonparametric data). Patient data collected before and after the course were compared using a Kruskal-Wallis test (for nonparametric numerical data) or chi-square test (for categorical data). A p value of <0.05 was considered significant, and 95% confidence intervals (CIs) were calculated.

## Results

A total of 88 open-fracture case files were identified across the 4 participating hospitals; 43 of the 88 injuries occurred before the guideline training course and were analyzed retrospectively, and 45 occurred after the course and were analyzed prospectively. Zomba Central Hospital treated 34% (30); Mangochi District Hospital, 24% (21); Mulanje District Hospital, 16% (14); and Salima District Hospital, 26% (23). The median age of the patients was 36 years (IQR, 27 to 45 years), and 91% (80) were male. There was no significant difference between the patient groups before and after the guideline training course with respect to age (p = 0.083), gender (p = 0.761), occupation (p = 0.632), comorbidity (p = 1.000), other associated injuries (p = 0.295), or median days from the injury to presentation at the hospital (p = 0.116) (Table I).

**TABLE I:** Baseline Demographics of Participants with an Open Fracture Before and After the Guidelines*

	Before (N = 43)	After (N = 45)	Overall (N = 88)	P Value
Age *(yr)*	31 (25-42)	40 (28-54)	36 (27-45)	0.083
Male	93% (40)	89% (40)	91% (80)	0.761
Other associated injuries†	5% (2)	13% (6)	9% (8)	0.295
Occupational status‡				0.632
Business	25% (2)	27% (6)	27% (8)	
Student	25% (2)	23% (5)	23% (7)	
Farmer	12% (1)	14% (3)	13% (4)	
Office work	25% (2)	5% (1)	10% (3)	
Unemployed	0% (0)	14% (3)	10% (3)	
Self-employed	0% (0)	9% (2)	7% (2)	
Minor	0% (0)	5% (1)	3% (1)	
Other	12% (1)	5% (1)	7% (2)	
Referred from§				0.07
Self-referral	68% (25)	56% (24)	61% (49)	
Health center	11% (4)	21% (9)	16% (13)	
District hospital	16% (6)	12% (5)	14% (11)	
Private clinic	0% (0)	12% (5)	6% (5)	
Christian Health Association of Malawi hospitals	5% (2)	0% (0)	2% (2)	
Days from injury to hospital presentation	0 (0-1)	0 (0-0)	0 (0-1)	0.116

* Values are given as the percentage of patients with the number in parentheses, except for age and days from injury to hospital, which are given as the median with the IQR in parentheses.

† Other associated injuries were defined as head injury, abdominal injury, chest injury, spinal injury, and closed long-bone fractures.

‡ 66% (58) of the patients had missing data.

§ 9% (8) of the patients had missing data.

The most common cause of the open fractures was an RTC, at 60% (53), and half of the patients in this group (49% [26]) had been riding a motorcycle at the time of the accident (Table II). The tibia was the most commonly fractured bone, at 67% (59).

**TABLE II:** Mechanisms of Injury*

	No. (%) of Patients
RTC	60% (53)
Motorcyclist	49% (26)
Pedestrian	26% (14)
Car driver	23% (12)
Bicyclist	2% (1)
Blunt trauma	11% (9)
Fall from greater than a standing height	10% (8)
Crocodile bite	7% (6)
Assault	5% (4)
Fall from a standing height	2% (2)
Sport	2% (2)

* 5% (4) of the patients had missing data. Values are given as the percentage of the remaining 84 patients with the number in parentheses, except for the breakdown of the individuals injured in RTCs, which are of the 53 with RTC injuries.

### Initial Assessment and Management of Open Fractures in the Emergency Department

The rate of ATLS assessment and documentation significantly improved (p = 0.029) from 60% (26) to 82% (37). There was also improvement in the percentage of patients who received neurovascular status assessment and documentation, from 26% (11) to 62% (28) (p = 0.0002), and for whom wound photography was performed before debridement, from 0% (0) to 51% (23) (p = 0.01). However, there was no significant difference in Gustilo-Anderson wound grading (p = 0.372), type of antibiotics (p = 0.588), temporary immobilization (p = 0.878), or median time to antibiotics (p = 0.103). Tetanus prophylaxis remained low in both phases, at 2% (1) before and 4% (2) after the guideline training course (p = 1) (Table III).

**TABLE III:** Initial Assessment and Management of Open Fractures Before and After the Guidelines*

	Before (N = 43)	After (N = 45)	P Value
ATLS assessment made and documented	60% (26)	82% (37)	0.029
Neurovascular status assessed and documented	26% (11)	62% (28)	0.0002
Gustilo wound grading			0.372
Not documented	42% (18)	26% (11)	
Type I	21% (9)	31% (14)	
Type II	16% (7)	13% (6)	
Type IIIA	16% (7)	13% (6)	
Type IIIB	5% (2)	13% (6)	
Type IIIC	0% (0)	4% (2)	
Radiograph	93% (40)	96% (43)	0.487
Time to antibiotics† *(hr)*	4.5 (1.25-42)	8 (1-24)	0.103
Types of antibiotics‡			0.588
Ceftriaxone	63% (27)	80% (36)	
Metronidazole	28% (12)	40% (18)	
Penicillin	16% (7)	22% (10)	
Flucloxacillin	21% (9)	13% (6)	
Gentamicin	7% (3)	7% (3)	
Doxycycline	7% (3)	4% (2)	
Amoxicillin	0% (0)	4% (2)	
Ciprofloxacin	0% (0)	4% (2)	
Not documented	14% (6)	7% (3)	
Tetanus toxoid vaccine	2% (1)	4% (2)	1
Temporary immobilization			0.878
POP slabs	65% (28)	71% (32)	
Skin traction	2% (1)	4% (2)	
None	12% (5)	11% (5)	
Bandage only	0% (0)	0% (0)	
External fixator	0% (0)	0% (0)	
Not documented	21% (9)	13% (6)	
Wound photography	0% (0)	51% (23)	0.01

* Values are given as the percentage of patients with the number in parentheses, except for time to antibiotics, which is given as the median with the IQR in parentheses.

† 56% (49) of the patients had missing data.

‡ 59% (52) of the patients received more than a single type of antibiotic

### Place and Type of Anesthesia for Open Fracture Treatment

**Fig. 1:**
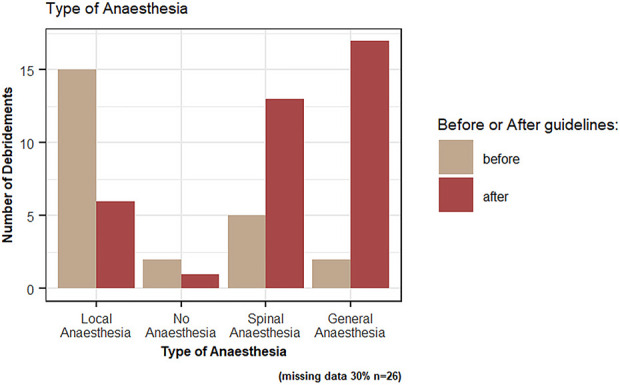
Types of anesthesia before and after the MOA/AOA open fracture guidelines.

The percentage of patients undergoing debridement in the operating room increased from 19% (8) to 69% (31) (p = 0.01). Similarly, the type of anesthesia changed (p = 0.001), with the percentage who had debridement under general or spinal anesthesia significantly increasing from 5% (2) to 38% (17) and from 12% (5) to 29% (13), respectively (Fig. 1).

### Definitive Treatment and Referral of Severe Open Fractures

There was no significant difference in the type of definitive treatment (p = 0.42); at least as many cases were treated with POP before 52% (16) and after 42% (15) the guideline training course than with other methods. Most of the definitive treatments were performed by OCOs in both phases, 58% (25) before and 78% (35) after the guideline implementation. There was also no significant difference in the median days to definitive treatment (p = 0.159) or in who performed the surgery (p = 0.065) between the groups. Additionally, the median days to referral remained the same, 1 day (IQR, 0 to 1 days) before and 1 day (IQR, 0 to 3 days) after the guideline training course. There was no significant improvement (p = 0.083) in the percentage who underwent open-fracture debridement before referral, 40% (4) before and 64% (7) after the guideline implementation (Table IV).

**TABLE IV:** Definitive Treatment and Complications Before and After the Guideline*

	Before (N = 43)	After (N = 45)	P Value
Definitive treatment†			0.42
POP	52% (16)	42% (15)	
External fixator	42% (13)	42% (15)	
Internal fixation	6% (2)	3% (1)	
Straight arm traction	0% (0)	3% (1)	
Skeletal traction	0% (0)	8% (3)	
Amputation	0% (0)	3% (1)	
Days to definitive treatment and debridement	0 (0-1)	1 (0-1.5)	0.159
Debridement and definitive treatment performed by			0.065
Orthopaedic clinical officer	58% (25)	78% (35)	
Orthopaedic surgeon	9% (4)	0% (0)	
Trainee orthopaedic clinical technician	2% (1)	0% (0)	
Surgical intern	0% (0)	4% (2)	
Other	0% (0)	2% (1)	
Not documented	26% (11)	9% (4)	
Referral site	5% (2)	7% (3)	
Complications‡			0.152
Wound infection	21% (9)	11% (5)	
Compartment syndrome	0% (0)	0% (0)	
Amputation	5% (2)	7% (3)	
Other	2% (1)	4% (2)	
Death	0% (0)	7% (3)	
Debridement before referral§	40% (4)	64% (7)	0.083
Days to referral	1 (0-1)	1 (0-3)	1

* Values are given as the percentage of patients with the number in parentheses, except for days to definitive treatment and days to referral, which are given as the median with the IQR in parentheses.

† 24% (21) were referred.

‡ 73% (64) of the patients did not develop any of the listed complications.

§ 48% (10) of 21 patients had missing data on debridement before referral.

### Complications

A total of 24 patients (27%) developed complications. The rate of wound infections was 21% (9) before the guideline training course and 11% (5) after. The rate of amputation was 5% (2) before and 7% (3) after. Both of the patients who underwent amputation in the before-guideline group had developed an uncontrollable infection; 2 of the 3 amputees in the after-guideline group had an avascular limb in the absence of vascular reconstruction and the third had a mangled extremity with the absence of reconstruction capabilities. No patient died before the implementation of the guidelines, whereas 7% (3) died after implementation due to tetanus, an unknown cause, and septicemia (2% [1] each). However, there was no significant difference (p = 0.152) in the overall rate of complications between before and after the guideline training course (Table IV).

## Discussion

This study showed an improvement in the management of open fractures after implementation of the MOA/AOA guidelines through a training course.

One surprising finding was that half of the patients in RTCs were motorcycle riders rather than pedestrians, contrary to previous studies done in Malawi^6,8^. This could be explained by the rise of the motorcycle taxi business in Malawi since 2020. Most of these motorcycles are not registered, and the drivers do not undergo certified driver’s training^29^. There is a need for the Ministry of Health, Directorate of Curative and Rehabilitation Services, Directorate of Road Traffic and Safety Services, Malawi Police Services, Kabaza Association of Malawi, and all stakeholders to devise strategies to sensitize the public regarding road traffic safety issues and reinforce road traffic laws.

The data indicate a significant improvement in the percentage of patients who underwent wound debridement in the operating room under general or spinal anesthesia (p = 0.001). This is contrary to a previous study that showed that general rather than regional anesthesia was commonly used before and after implementation of the open-fracture-treatment guideline^30^. The difference may be because our study was performed at a combination of central and district hospitals. Most district hospitals have only 1 main operating room to treat all conditions that require surgery (obstetrics, general surgery, and orthopaedics) and 1 minor operating room in the emergency department for minor emergencies^19^. Obstetric cases are typically prioritized in the main operating room, in accordance with Malawi’s Safe Motherhood Initiative program^19,31,32^. However, studies have shown that when a patient with an open fracture is fully anesthetized, a more thorough debridement can be achieved, which may reduce the possibility of infection^33,34^. A need for good teamwork and coordination between anesthesia, obstetrics and gynecology, surgery, and orthopaedics is crucial for optimizing use of operating room space within limited resources.

The facts that POP remained the definitive open fracture treatment for almost half of patients both before and after guideline implementation, with delays when severe injuries (Gustilo-Anderson IIIA and IIIB) were referred to central hospitals, can be explained by the ready availability of POP and OCOs in most district hospitals and inadequate ambulance services to transfer patients to central hospitals^35-38^. This is similar to previous studies in Malawi, but studies in Nigeria and southwest Cameroon have shown that open fractures were initially treated with external fixation or intramedullary nailing and reconstructive surgery by orthopaedic and plastic surgeons^15,39-41^. Immediate referral of severe open fractures to central hospitals for orthopaedic and plastic interventions might improve patients’ outcomes, but further study is required to understand the effect and feasibility of immediate referral^15,41^. There is also a need to develop clear referral protocols to improve the referral system for severe open fractures to central hospitals for nailing and early soft-tissue coverage as well as for increasing orthopaedic and plastic surgeon capacity for district hospitals^25^.

There was no significant difference in the overall complication rate between the 2 groups (p = 0.152. The infection rate in our study is similar to the approximately 18% rate in studies from the U.K. and from other LMICs^9,15^. Improving surgical expertise in soft-tissue management might help reduce infection rates, particularly in terms of wound closure and complete debridements^42^. Many LMICs, including Malawi, have very limited plastic surgeon availability for open fractures^43^. During the study, there were only 2 plastic surgeons working in government hospitals in Malawi, neither of whom was available in the study sites^21^. Additionally, 13% of the patients treated after guideline implementation were diagnosed with a Gustilo-Anderson type-IIIB injury, which could have equally benefited from soft-tissue reconstructive surgery (Table III). These open fractures should be treated with early antibiotics and safe and adequate debridement to reduce the risk of infection, and greater plastic surgeon availability at tertiary hospitals is needed to provide early soft-tissue coverage^25^.

The administration of tetanus toxoid vaccine (TTV) prophylaxis remained at a low rate of 4%, which stands in stark contrast to the findings of Mkandawire et al., who reported an increase to 95% after implementation of the open fracture guideline compared to 60% before the guideline^32^. The difference in TTV prophylaxis rates might be attributable to the recent demand for TTV, particularly among pregnant women and women of reproductive age, as part of the Expanded Programme on Immunization (EPI)^44^. As a result, the priority for TTV administration is primarily pregnant women, with less emphasis placed on patients with open fractures^44^. One of the recorded deaths in our study was attributed to tetanus infection; therefore, future research initiatives and ongoing implementation efforts involving government policymakers should emphasize the importance of TTV administration for patients with open fractures.

### Limitations

Overall, the rate of missing data was 25% because part of the study was retrospective. Furthermore, data capture was manual. This contributed to the high rate of missing data for important variables such as definitive treatment, wound closure, and infection rate, which had the potential to bias the stratification of the results according to the Gustilo-Anderson classification. Implementing direct electronic data entry could help minimize missing data in future studies.

We refrained from stratifying infections on the basis of the mechanism of injury, such as crocodile bites. Crocodile bites pose a considerable challenge and often result in complex open fracture injuries with infections stemming from a variety of oral microbes associated with these incidents. We therefore did not perform analyses according to the injury mechanism to prevent any potential impact on the interpretation of the value of early surgical intervention for the remaining cases^45^. The current literature on animal bites emphasizes the variability in risks, management algorithms, and potential complications associated with different types of bites, including crocodile bites^46^. Despite these differences, there is a consensus that prompt intervention is vital for alleviating symptoms and reducing complications^46^. A further comprehensive review and synthesis of the existing literature is needed to shed light on the fundamentals of the treatment of open fractures resulting from crocodile bites.

Furthermore, long-term outcomes were not analyzed in this study and remain unknown. Short to long-term outcomes and complications of open fractures can include infection, amputation, compartment syndrome, nonunion, malunion, and chronic osteomyelitis. Future studies should also focus on the long-term outcomes of patients with open fractures. The addition of infographics on open fracture guidelines and temporary patient note recording forms as part of the implementation package during the training might have made the training course more effective and at least temporarily improved the management of open fractures. There is also a need for additional studies to evaluate retention of the gained knowledge on open fracture management over time.

### Conclusions

This study suggests that the training course on the MOA/AOA guidelines in open-fracture management combined with the practice of these evidence-based guidelines can lead to at least short-term improvement in the management of open fractures and significantly contributes to the improved documentation of open fracture treatment. However, it is essential to acknowledge that our study primarily focused on short-term outcomes. Additional in-depth studies are warranted to establish a more comprehensive understanding of the long-term outcomes as well as perform a broader exploration of potentially influential factors, such as the mechanism of injury.

## Appendix

Supporting material provided by the authors is posted with the online version of this article as a data supplement at jbjs.org (http://links.lww.com/JBJSOA/A614).
